# Supplementary material for: Effects of Golimumab and Ustekinumab on Circulating Dendritic Cell Migratory Capacity in Inflammatory Bowel Disease
Source: Biomedicines. 2023 Oct 18;11(10):2831. doi: 10.3390/biomedicines11102831 (PMC10603850; doi:10.3390/biomedicines11102831)
Supplement: Supplementary file 1 [file biomedicines-11-02831-s001.zip › biomedicines-2644527-supplementary.pptx]

## Slide 1
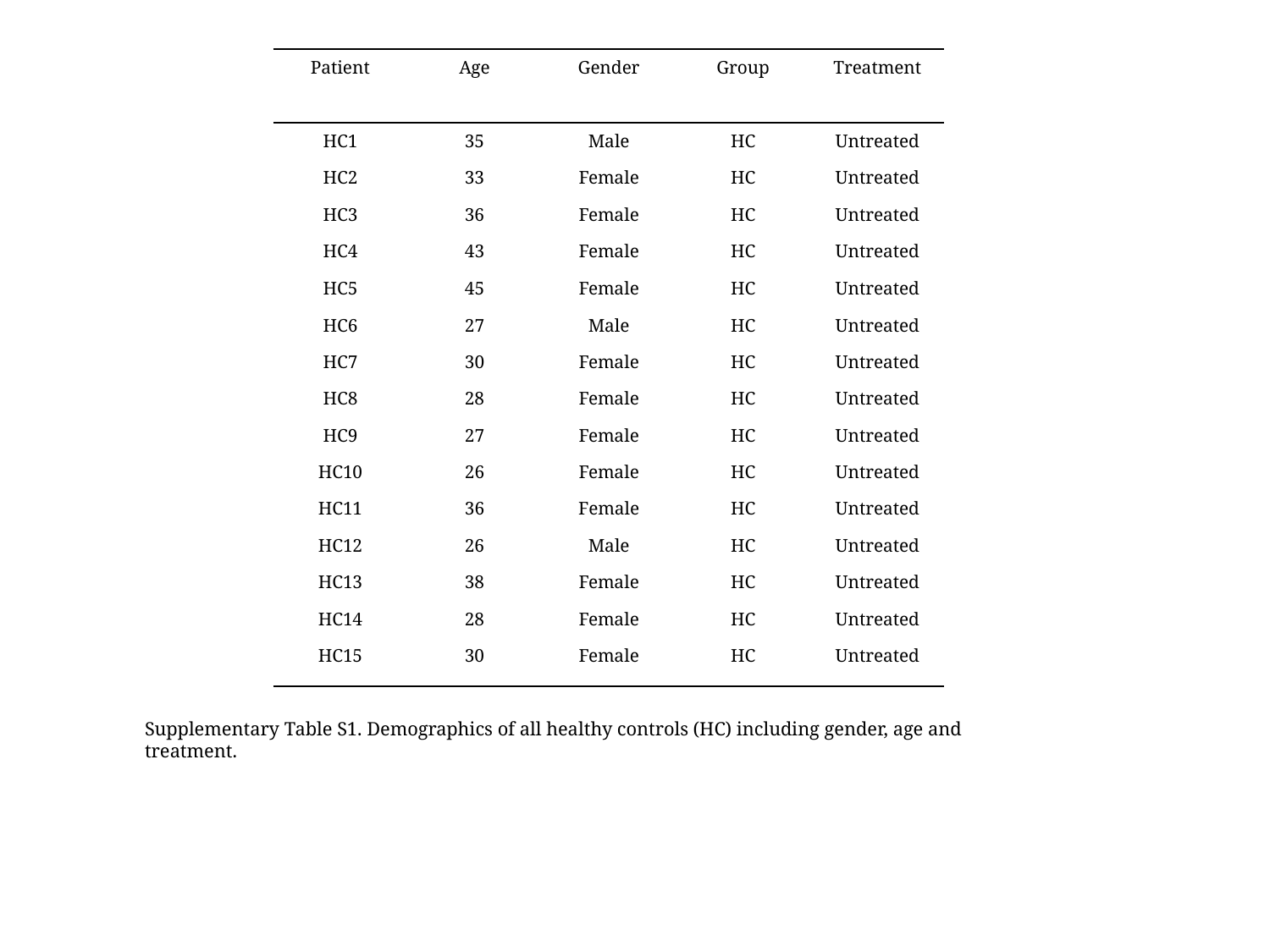

| Patient | Age | Gender | Group | Treatment |
| --- | --- | --- | --- | --- |
| | | | | |
| HC1 | 35 | Male | HC | Untreated |
| HC2 | 33 | Female | HC | Untreated |
| HC3 | 36 | Female | HC | Untreated |
| HC4 | 43 | Female | HC | Untreated |
| HC5 | 45 | Female | HC | Untreated |
| HC6 | 27 | Male | HC | Untreated |
| HC7 | 30 | Female | HC | Untreated |
| HC8 | 28 | Female | HC | Untreated |
| HC9 | 27 | Female | HC | Untreated |
| HC10 | 26 | Female | HC | Untreated |
| HC11 | 36 | Female | HC | Untreated |
| HC12 | 26 | Male | HC | Untreated |
| HC13 | 38 | Female | HC | Untreated |
| HC14 | 28 | Female | HC | Untreated |
| HC15 | 30 | Female | HC | Untreated |
Supplementary Table S1. Demographics of all healthy controls (HC) including gender, age and treatment.

## Slide 2
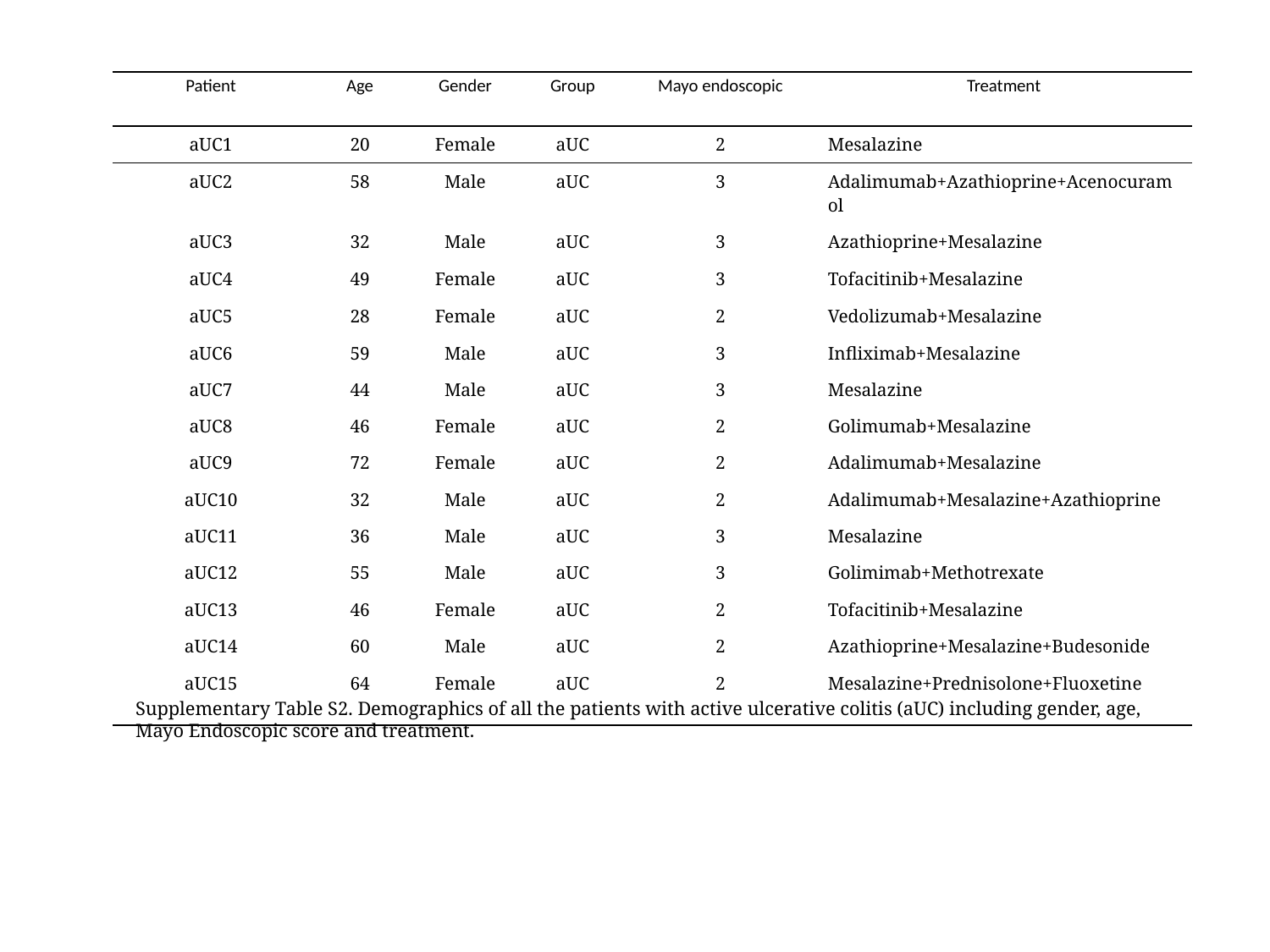

| Patient | Age | Gender | Group | Mayo endoscopic | Treatment |
| --- | --- | --- | --- | --- | --- |
| aUC1 | 20 | Female | aUC | 2 | Mesalazine |
| aUC2 | 58 | Male | aUC | 3 | Adalimumab+Azathioprine+Acenocuramol |
| aUC3 | 32 | Male | aUC | 3 | Azathioprine+Mesalazine |
| aUC4 | 49 | Female | aUC | 3 | Tofacitinib+Mesalazine |
| aUC5 | 28 | Female | aUC | 2 | Vedolizumab+Mesalazine |
| aUC6 | 59 | Male | aUC | 3 | Infliximab+Mesalazine |
| aUC7 | 44 | Male | aUC | 3 | Mesalazine |
| aUC8 | 46 | Female | aUC | 2 | Golimumab+Mesalazine |
| aUC9 | 72 | Female | aUC | 2 | Adalimumab+Mesalazine |
| aUC10 | 32 | Male | aUC | 2 | Adalimumab+Mesalazine+Azathioprine |
| aUC11 | 36 | Male | aUC | 3 | Mesalazine |
| aUC12 | 55 | Male | aUC | 3 | Golimimab+Methotrexate |
| aUC13 | 46 | Female | aUC | 2 | Tofacitinib+Mesalazine |
| aUC14 | 60 | Male | aUC | 2 | Azathioprine+Mesalazine+Budesonide |
| aUC15 | 64 | Female | aUC | 2 | Mesalazine+Prednisolone+Fluoxetine |
Supplementary Table S2. Demographics of all the patients with active ulcerative colitis (aUC) including gender, age, Mayo Endoscopic score and treatment.

## Slide 3
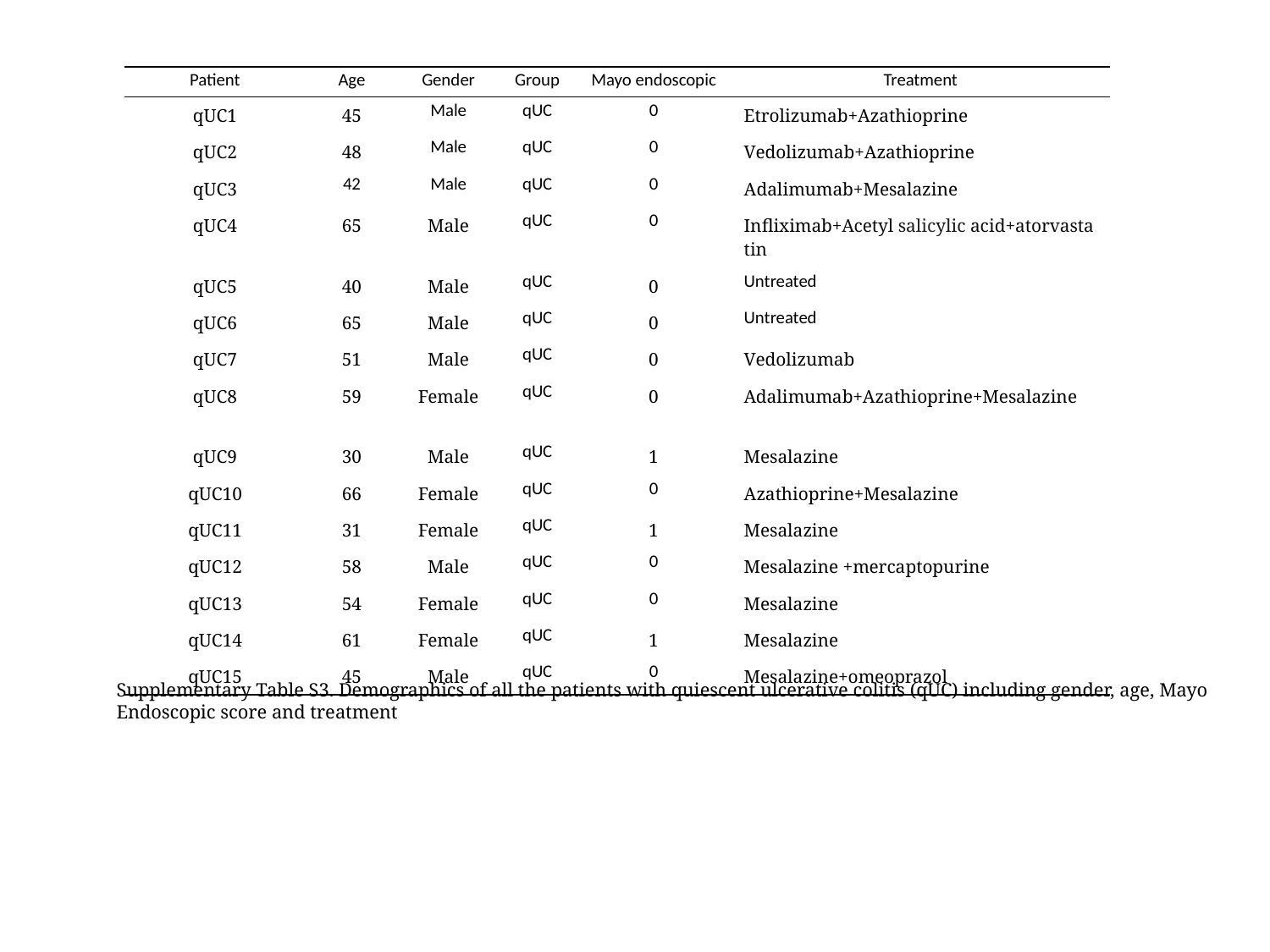

| Patient | Age | Gender | Group | Mayo endoscopic | Treatment |
| --- | --- | --- | --- | --- | --- |
| qUC1 | 45 | Male | qUC | 0 | Etrolizumab+Azathioprine |
| qUC2 | 48 | Male | qUC | 0 | Vedolizumab+Azathioprine |
| qUC3 | 42 | Male | qUC | 0 | Adalimumab+Mesalazine |
| qUC4 | 65 | Male | qUC | 0 | Infliximab+Acetyl salicylic acid+atorvastatin |
| qUC5 | 40 | Male | qUC | 0 | Untreated |
| qUC6 | 65 | Male | qUC | 0 | Untreated |
| qUC7 | 51 | Male | qUC | 0 | Vedolizumab |
| qUC8 | 59 | Female | qUC | 0 | Adalimumab+Azathioprine+Mesalazine |
| qUC9 | 30 | Male | qUC | 1 | Mesalazine |
| qUC10 | 66 | Female | qUC | 0 | Azathioprine+Mesalazine |
| qUC11 | 31 | Female | qUC | 1 | Mesalazine |
| qUC12 | 58 | Male | qUC | 0 | Mesalazine +mercaptopurine |
| qUC13 | 54 | Female | qUC | 0 | Mesalazine |
| qUC14 | 61 | Female | qUC | 1 | Mesalazine |
| qUC15 | 45 | Male | qUC | 0 | Mesalazine+omeoprazol |
Supplementary Table S3. Demographics of all the patients with quiescent ulcerative colitis (qUC) including gender, age, Mayo Endoscopic score and treatment

## Slide 4
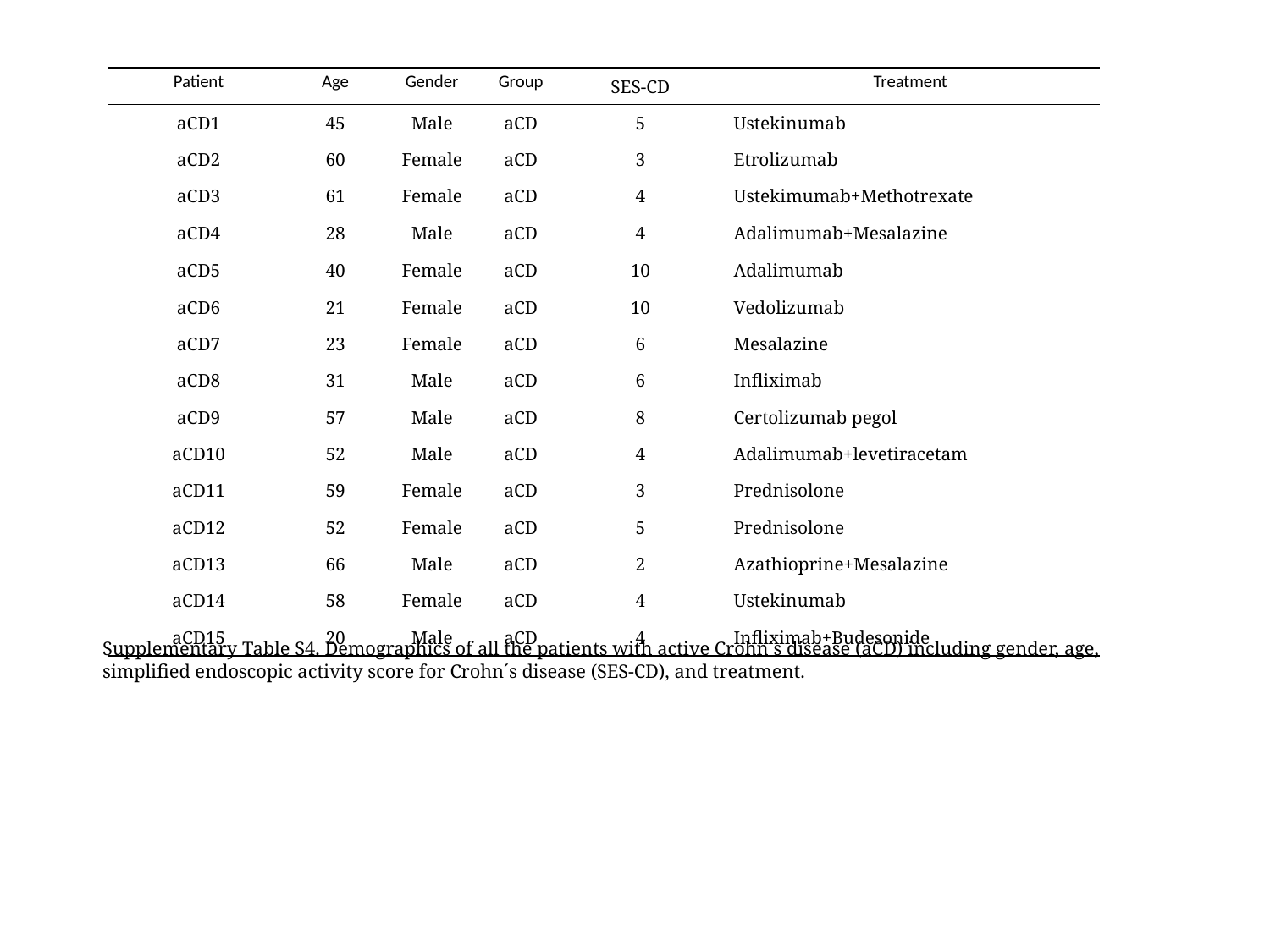

| Patient | Age | Gender | Group | SES-CD | Treatment |
| --- | --- | --- | --- | --- | --- |
| aCD1 | 45 | Male | aCD | 5 | Ustekinumab |
| aCD2 | 60 | Female | aCD | 3 | Etrolizumab |
| aCD3 | 61 | Female | aCD | 4 | Ustekimumab+Methotrexate |
| aCD4 | 28 | Male | aCD | 4 | Adalimumab+Mesalazine |
| aCD5 | 40 | Female | aCD | 10 | Adalimumab |
| aCD6 | 21 | Female | aCD | 10 | Vedolizumab |
| aCD7 | 23 | Female | aCD | 6 | Mesalazine |
| aCD8 | 31 | Male | aCD | 6 | Infliximab |
| aCD9 | 57 | Male | aCD | 8 | Certolizumab pegol |
| aCD10 | 52 | Male | aCD | 4 | Adalimumab+levetiracetam |
| aCD11 | 59 | Female | aCD | 3 | Prednisolone |
| aCD12 | 52 | Female | aCD | 5 | Prednisolone |
| aCD13 | 66 | Male | aCD | 2 | Azathioprine+Mesalazine |
| aCD14 | 58 | Female | aCD | 4 | Ustekinumab |
| aCD15 | 20 | Male | aCD | 4 | Infliximab+Budesonide |
Supplementary Table S4. Demographics of all the patients with active Crohn´s disease (aCD) including gender, age, simplified endoscopic activity score for Crohn´s disease (SES-CD), and treatment.

## Slide 5
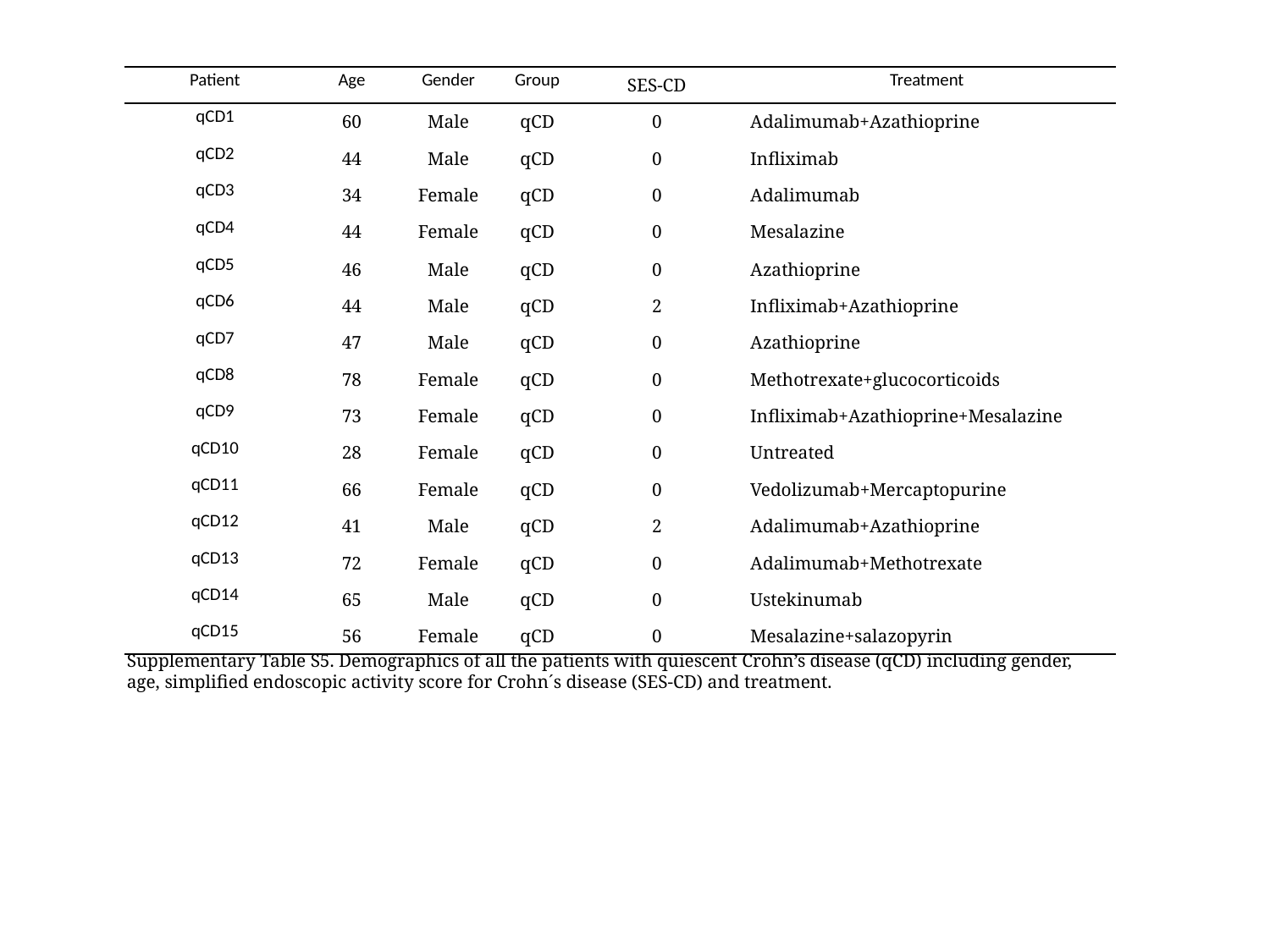

| Patient | Age | Gender | Group | SES-CD | Treatment |
| --- | --- | --- | --- | --- | --- |
| qCD1 | 60 | Male | qCD | 0 | Adalimumab+Azathioprine |
| qCD2 | 44 | Male | qCD | 0 | Infliximab |
| qCD3 | 34 | Female | qCD | 0 | Adalimumab |
| qCD4 | 44 | Female | qCD | 0 | Mesalazine |
| qCD5 | 46 | Male | qCD | 0 | Azathioprine |
| qCD6 | 44 | Male | qCD | 2 | Infliximab+Azathioprine |
| qCD7 | 47 | Male | qCD | 0 | Azathioprine |
| qCD8 | 78 | Female | qCD | 0 | Methotrexate+glucocorticoids |
| qCD9 | 73 | Female | qCD | 0 | Infliximab+Azathioprine+Mesalazine |
| qCD10 | 28 | Female | qCD | 0 | Untreated |
| qCD11 | 66 | Female | qCD | 0 | Vedolizumab+Mercaptopurine |
| qCD12 | 41 | Male | qCD | 2 | Adalimumab+Azathioprine |
| qCD13 | 72 | Female | qCD | 0 | Adalimumab+Methotrexate |
| qCD14 | 65 | Male | qCD | 0 | Ustekinumab |
| qCD15 | 56 | Female | qCD | 0 | Mesalazine+salazopyrin |
Supplementary Table S5. Demographics of all the patients with quiescent Crohn’s disease (qCD) including gender, age, simplified endoscopic activity score for Crohn´s disease (SES-CD) and treatment.

## Slide 6
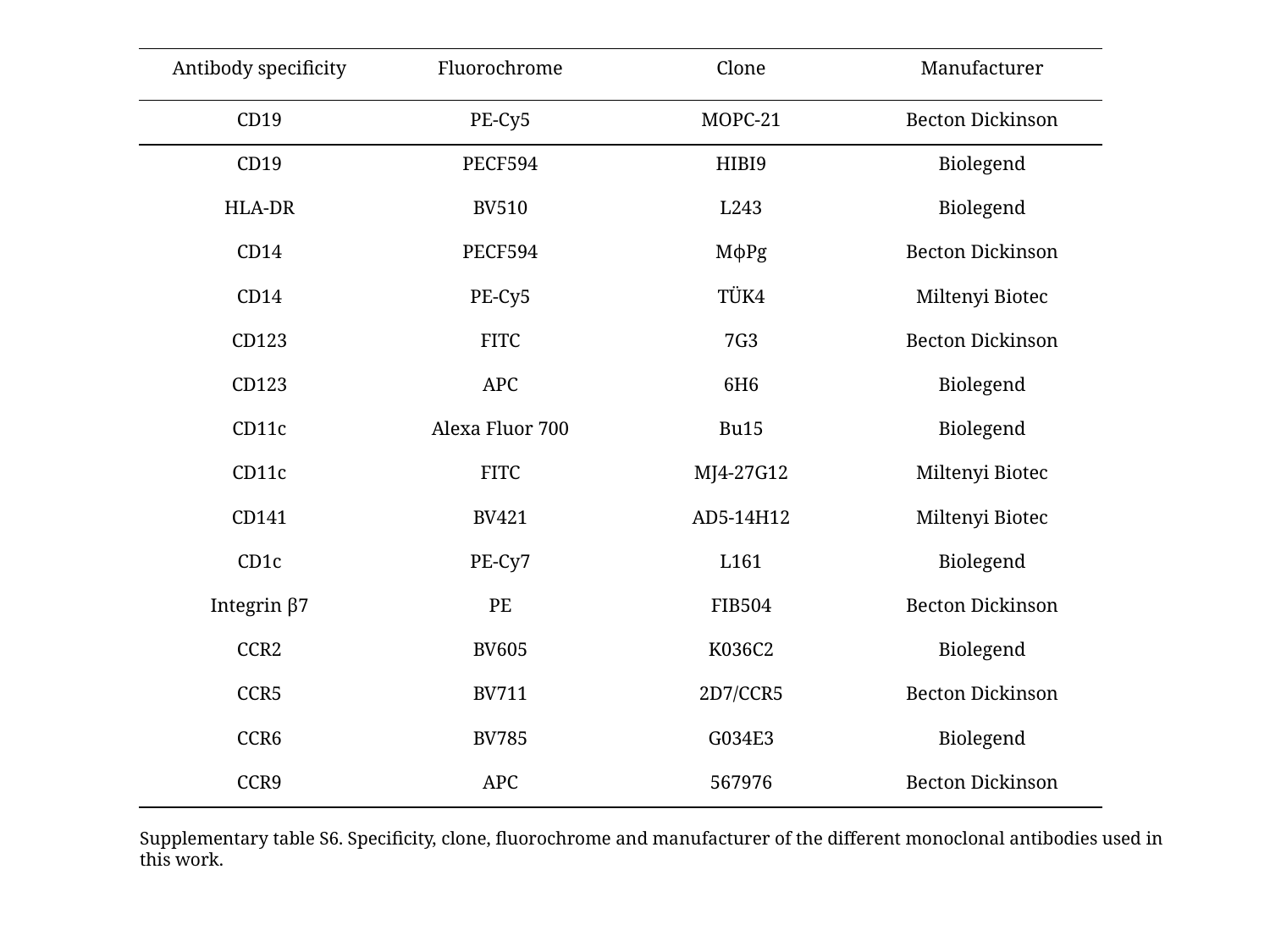

| Antibody specificity | Fluorochrome | Clone | Manufacturer |
| --- | --- | --- | --- |
| CD19 | PE-Cy5 | MOPC-21 | Becton Dickinson |
| CD19 | PECF594 | HIBI9 | Biolegend |
| HLA-DR | BV510 | L243 | Biolegend |
| CD14 | PECF594 | MϕPg | Becton Dickinson |
| CD14 | PE-Cy5 | TÜK4 | Miltenyi Biotec |
| CD123 | FITC | 7G3 | Becton Dickinson |
| CD123 | APC | 6H6 | Biolegend |
| CD11c | Alexa Fluor 700 | Bu15 | Biolegend |
| CD11c | FITC | MJ4-27G12 | Miltenyi Biotec |
| CD141 | BV421 | AD5-14H12 | Miltenyi Biotec |
| CD1c | PE-Cy7 | L161 | Biolegend |
| Integrin β7 | PE | FIB504 | Becton Dickinson |
| CCR2 | BV605 | K036C2 | Biolegend |
| CCR5 | BV711 | 2D7/CCR5 | Becton Dickinson |
| CCR6 | BV785 | G034E3 | Biolegend |
| CCR9 | APC | 567976 | Becton Dickinson |
Supplementary table S6. Specificity, clone, fluorochrome and manufacturer of the different monoclonal antibodies used in this work.
